# Lipoprotein(a) is Markedly More Atherogenic than LDL: An Apolipoprotein B-based Genetic Analysis

**DOI:** 10.1016/j.jacc.2023.10.039

**Published:** 2024-01-23

**Authors:** Elias Björnson, Martin Adiels, Marja-Riitta Taskinen, Stephen Burgess, M. John Chapman, Chris J Packard, Jan Borén

**Affiliations:** a Department of Molecular and Clinical Medicine, University of Gothenburg, Gothenburg, Sweden; b School of Public Health and Community Medicine, Institute of Medicine, University of Gothenburg, Gothenburg, Sweden; c Research Program for Clinical and Molecular Metabolism, University of Helsinki; University of Helsinki, Finland; d MRC Biostatistics Unit, University of Cambridge, Cambridge, UK; e Cardiovascular Epidemiology Unit, Department of Public Health and Primary Care, University of Cambridge, Cambridge, UK; f Faculty of Medicine, Sorbonne University, and Cardiovascular Disease Prevention Unit, Pitie-Salpetriere Hospital, Paris, France; g Institute of Cardiovascular and Medical Sciences, University of Glasgow, Glasgow, UK

**Keywords:** Mendelian randomisation, cardiovascular disease, lipoprotein (a), LDL cholesterol, UK Biobank

## Abstract

**Background and Aims:**

Lipoprotein (a) (Lp(a)) is recognised as a causal factor for coronary heart disease (CHD) but its atherogenicity relative to that of LDL on a per particle basis is indeterminate.

**Objectives:**

We addressed this issue in a genetic analysis based on the fact that Lp(a) and LDL both contain one apolipoprotein B per particle.

**Methods:**

Genome wide association studies using the UK Biobank population identified two clusters of SNPs: one comprising 107 variants linked to Lp(a) mass concentration, the other with 143 variants linked to LDL concentration. In these ‘Lp(a)’ and ‘LDL’ clusters, the relationship of genetically-predicted variation in apoB with CHD risk was assessed.

**Results:**

The Mendelian randomisation-derived odds ratio for CHD for a 50 nmol/L higher Lp(a)-apoB was 1.28 [95% confidence interval (CI):1.24-1.33] compared to 1.04 [95%CI:1.03-1.05] for the same increment in LDL-apoB. Likewise, use of polygenic scores to rank subjects according to difference in Lp(a)-apoB vs. difference in LDL-apoB revealed a greater hazard ratio for CHD per 50nmol/l apoB for the ‘Lp(a)’ cluster (1.47 [95%CI:1.36-1.58]) compared to the ‘LDL’ cluster (1.04 [95%CI:1.02-1.05]). From these data, we estimate that the atherogenicity of Lp(a) is around 6-fold [point estimate of 6.6; 95% CI:5.1-8.8] greater than that of LDL on a per particle basis.

**Conclusions:**

We conclude that the atherogenicity of Lp(a) (coronary heart disease risk quotient per unit increase in particle number) is substantially greater than that of LDL. Therefore, Lp(a) represents a key target for drug-based intervention in a significant proportion of the at-risk population.

## Graphical abstract

**Graphical Abstract:**
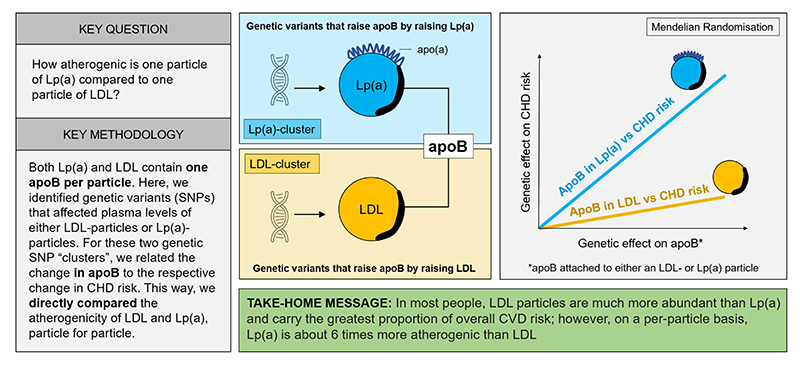

## Introduction

Lipoprotein (a) (Lp(a)) - an LDL-like particle containing apolipoprotein B (apoB) with an additional protein, apolipoprotein (a) (apo(a)) - has been identified as a causal factor for coronary heart disease (CHD) based initially on epidemiological findings and most recently on genetic studies ^1–3^. Variants in the *LPA* gene coding for the apo(a) component of the lipoprotein have been associated with risk of myocardial infarction, stroke, and aortic valve stenosis ^1, 4^. The plasma concentration of Lp(a) is regulated primarily by genetic factors and varies widely within populations and between ethnic groups ^1^. Recently, Lp(a) has received attention as a potential target for drug-based intervention ^5^. Statins do not lower Lp(a) and may even increase moderately the level of this lipoprotein ^1^. Use of proprotein convertase subtilisin/kexin type 9 (PCSK9) inhibitors, in addition to lowering LDL cholesterol (LDL-C) decreases Lp(a), and this action has been reported to contribute to the reduction in CHD endpoints seen in clinical trials ^6, 7^. Specific agents to lower Lp(a) profoundly (>85%) are in clinical development. These RNA-based drugs act to suppress synthesis of the apo(a) polypeptide in the liver and thus inhibit Lp(a) formation ^5, 8–10^.

The magnitude of the benefit in terms of CHD reduction that would accompany targeted Lp(a) lowering is as yet unclear. A number of investigations, based on the relationship of genetically-predicted variation in Lp(a) to CHD risk, have yielded answers that vary considerably ^11–14^, and indicate that Lp(a) mass concentration would need to be lowered by 50-100 mg/dL to achieve the same risk reduction as a 1.0 mmol/L lower LDL cholesterol (LDL-C). Several factors may be responsible for uncertainty in determining the gradient of association of Lp(a) levels with CHD risk, one of which is the accuracy of measurement of its plasma concentration. Many assays are hampered by calibration issues, the isoform dependency of the test procedure (apo(a) can vary in length depending on the number of kringle-4 units present in the protein), and the fact that most individuals are heterozygous for Lp(a) isoforms, all of which may contribute to the challenge of assessing genetic variant effect sizes on Lp(a) ^15, 16^.

The present investigation focuses not on the apo(a) protein that is unique to Lp(a) particles but rather on their constituent apoB. Since Lp(a) is formed by the covalent addition of apo(a) to apoB on LDL particles during the hepatic secretory process, it is conceptually possible to determine the number of circulating Lp(a) particles by assessing the contained apoB. ApoB is present in other lipoproteins, but by identifying genetic variants that uniquely affect Lp(a) levels, it should be possible to quantify genetically driven differences in apoB in Lp(a). A similar approach can be used to quantify genetically driven changes in apoB in LDL by using variants uniquely related to LDL. We hypothesised that the relationship of “Lp(a)-apoB” and “LDL-apoB” to risk of CHD, would permit a comparison of the relative atherogenicity of these two lipoprotein species. We further hypothesised that this approach would provide insight into the expected CHD risk reduction from a given lowering of Lp(a).

## Methods

### Study population

This study was based principally on the UK Biobank population (over 502,000 UK residents of mainly European ancestry) ^17^ (Online Figure 1). A replication cohort, the CARDIoGRAMplusC4D data set, was used to test the generalisability of the findings ^18^. The study was conducted under the UK Biobank Resource application number 53308. Thus, the study is exempt from ethical review board approval.

### Rationale

This study takes advantage of the fact that it is possible to estimate the genetically driven variation in apoB in Lp(a) by first identifying SNPs that are associated with variation in Lp(a) mass concentration (as measured biochemically) and then determining the effect size (beta coefficients) of these Lp(a) specific SNPs on plasma apoB levels. See **Online Expanded Rationale and Methodology** for a more detailed presentation of the experimental approach used in this study. In the present paper the terms “apoB in Lp(a)” and “Lp(a)-apoB” refer to the genetically predicted effect of SNPs in the ‘Lp(a)’ cluster on total apoB. Similarly, the terms “apoB in LDL” and “LDL-apoB” refer to the genetically predicted effect of the SNPs in the ‘LDL’ cluster on total apoB. ApoB was not measured in the Lp(a) or LDL fractions.

### Lipid measurements

LDL-C was measured directly (Beckman Coulter, Brea, CA). TRL/remnant-C was derived by subtracting LDL-C and high-density lipoprotein cholesterol from total plasma cholesterol ^19^. Lipoprotein (a) mass concentration was measured in 377,572 participants by immunoturbidimetry using the Randox assay (Data Field 30790). The assay range was 3.8 to 189 nmol/L (for the purposes of the present investigation values outside the assay working range were classified ‘not available’). Assays for Lp(a) report concentration in mg/dL or in nmol/L depending on the calibration approach used. In this paper we use nmol/L and, where required, a conversion factor of 1mg/dL = 2.2 nmol/L which is based on a report where both assay calibrations (nmol/L and mg/dL) were compared ^13^. This factor is recognised as an approximation since there is no single molecular weight for Lp(a) ^20^. All other analytes were measured by standard laboratory methods (https://biobank.ctsu.ox.ac.uk). ApoB is reported in g/L in the UK Biobank data repository but for the purposes of the present investigation plasma concentrations were converted to nmol/L using a molecular weight of 550 kDa ^21^.

### Genetic analyses

Genotyping with the UK BiLEVE Axiom or UK Biobank Axiom arrays provided an evaluation of 805,426 single nucleotide polymorphisms (SNPs) spanning the entire genome (Online Figure 1).

#### Genome-wide association studies

Two GWAS adjusted for age, sex and genomic principal components 1-5 were conducted. SNPs meeting the significance threshold of <5×10^-8^ were pruned for linkage disequilibrium (r^2^ <0.1) and minor allele frequency (threshold >0.01). (see Online Figure 1 for a CONSORT-style diagram). In the first GWAS, SNPs associated with variation in plasma Lp(a) mass concentration (Randox assay) were identified. This SNP set was filtered for genetic variants that had effects on other lipoproteins, and the final group of SNPs designated the ‘Lp(a)’ cluster. In the second GWAS, SNPs were selected initially on the basis of variants having a significant association with plasma triglyceride, TRL/remnant-C and/or LDL-C but not Lp(a). This SNP set was filtered by excluding SNPs that gave an effect size ratio for TRL/remnant-C vs. LDL-C that exceeded 0.15 in order to minimise the contribution of remnant particles to the relationship of genetically-predicted LDL-C to CHD ^1^. The final SNP set was designated the ‘LDL’ cluster. (See Online Table 2 for examples of SNPs in each cluster with the highest effect sizes).

#### Generation of gene scores

Polygenic scores (PGS) for each SNP cluster - ‘Lp(a)’ and ‘LDL’ - were generated by first identifying the apoB raising allele for each SNP as the exposure allele. Each SNP was then weighted by the effect size on apoB and for each subject a score calculated as the weighted sum of the number of apoB raising alleles present. For each cluster, the entire cohort was then divided into 20^ths^ (ventiles) of PGS and mean levels of lipoprotein variables (apoB, LDL-C, Lp(a)) determined.

### CHD outcomes

These are defined in Online Table 1. For studies of the association of CHD with genetically predicted lipoprotein levels, outcomes were the combination of prevalent and incident events (myocardial infarction and coronary revascularisation). For the polygenic score studies, outcomes were incident events occurring during the approximately 12-year follow up period.

### Statistical methods and interpretation of genetic findings

All statistical analyses were performed using R version 4.0.4. Mendelian randomisation (MR) analyses based on the inverse variance-weighted (IVW) method (which assumes all variants are ‘valid’ instrumental variables; that is the SNP effect on CHD outcome is solely through its effect on the exposure/risk factor ^22^) were undertaken to determine the genetic relationship between apoB and CHD outcome for each cluster. Genetic instruments and beta coefficients were derived using data from subjects who had the required plasma lipoprotein levels available and were not on lipid-lowering therapy at baseline. Associations with CHD outcome were determined using data from all subjects (including those on treatment). Odds ratios for CHD outcomes were determined per 50 nmol/L higher apoB.

Polygenic gene scores (PGS) were formulated as described above and used to provide an assessment for the ‘Lp(a)’ cluster and the ‘LDL’ cluster of the relationships between variation in Lp(a)-apoB, and variation in LDL-apoB, and CHD risk. Cox proportional hazards models were fitted using CHD incidence data as outcome. The hazard ratio for each PGS term was scaled (according to the relationship between each PGS and plasma apoB) to calculate the hazard ratio per 50 nmol/L higher apoB.

### Definition of lipoprotein atherogenicity

The term ‘atherogenicity’ used throughout the manuscript refers to the difference in CHD risk per unit difference in Lp(a) or LDL particle number (molar concentration). We also propose adoption of the term ‘CHD risk quotient’ defined as the CHD relative risk associated with a unit difference in genetically predicted Lp(a)-apoB divided by the CHD relative risk associated with the same difference in genetically predicted LDL-apoB. The relative risk calculation is based on (log) odds ratios and the difference we have chosen to use is 50 nmol/L of apoB.

## Results

The subjects of this study consisted of 502,413 subjects of the UK Biobank cohort (54.4% female) in whom the appropriate SNP results were available. The mean LDL-C was 3.56 mmol/l, mean apoB was 1.03 g/L (1870 nmol/L), and mean Lp(a) was 44.6 nmol/L (median 21.2 nmol/L).

### Characteristics of SNPs in ‘Lp(a)’ and ‘LDL’ clusters

The GWAS analyses generated SNP clusters with the required features. After selection and pruning (Online Figure 1) the ‘Lp(a)’ cluster included 107 SNPs, all located in the region around the *LPA* gene, while the ‘LDL’ cluster included 143 SNPs which were at more widely dispersed loci. For each cluster example SNPs with the largest effect sizes are listed in Online Table 2.

**Figure 1:**
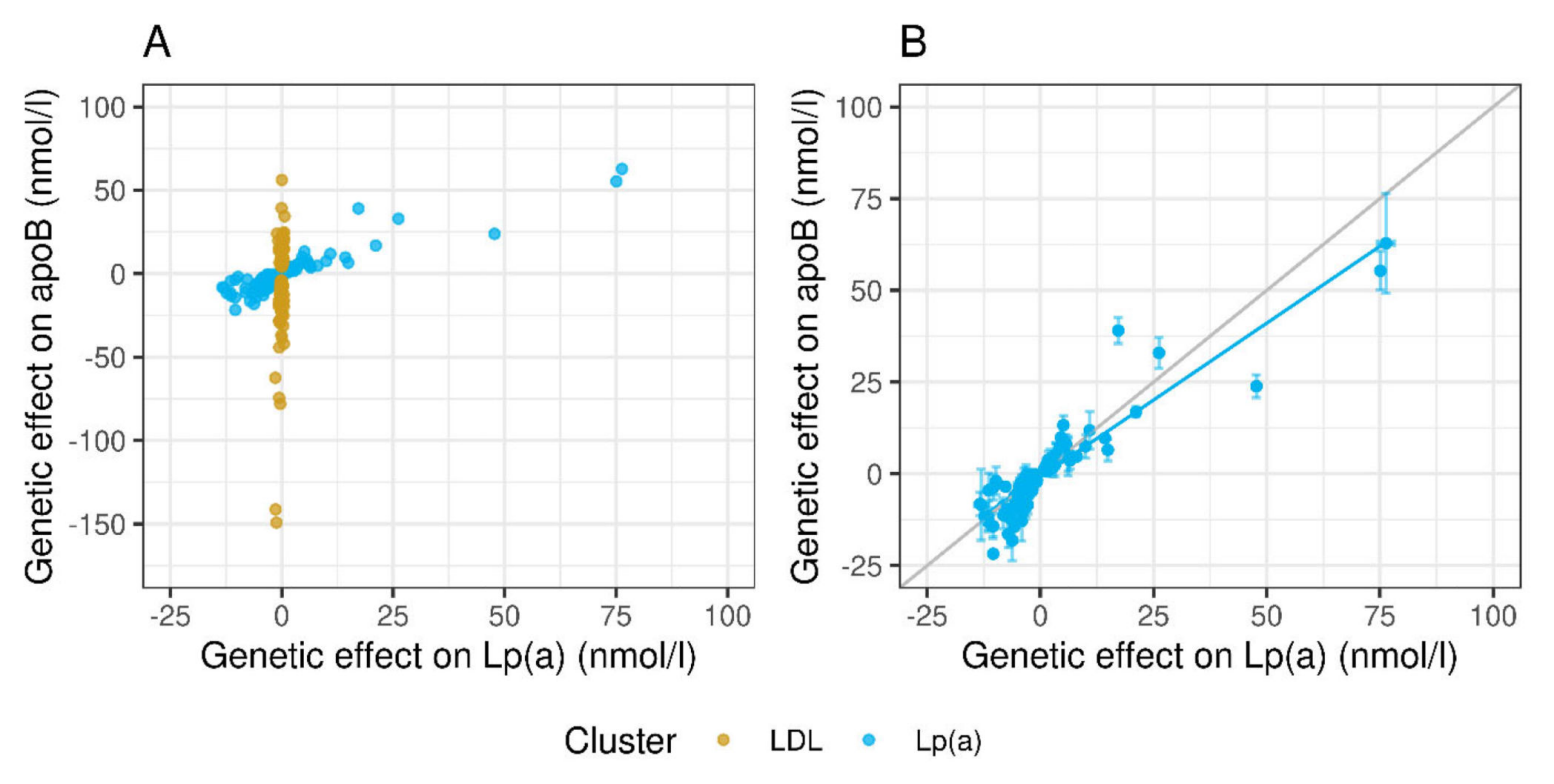
Effects of SNP clusters on plasma apoB and on Lp(a) levels. **Panel A:** Relationship of effect sizes (beta coefficients*) for plasma apoB and Lp(a) mass concentration for ‘Lp(a)’ cluster (blue) and ‘LDL’ SNP cluster (yellow). SNPs in the Lp(a) cluster affected both Lp(a) and apoB; SNPs in the ‘LDL’ cluster affected apoB but not Lp(a). **Panel B** Quantitative association of genetically predicted variation in Lp(a) mass concentration with Lp(a)-apoB (both in nmol/l) for ‘Lp(a)’ cluster. The two variables show good agreement, with the regression line near to unity. Thus, the selection criteria were successful in identifying SNP sets with the required properties. * Each data point represents the calculated beta-coefficient (and standard error) for each SNP. A beta coefficient is the mean change in the exposure (plasma levels of Lp(a) or apoB) in a population resulting from that population having one extra effect-allele. For example, a plasma apoB beta-coefficient for SNP X of 50 nmol/l means that heterozygotes for SNP X have, on average, 50 nmol/l higher apoB compared to people without the SNP. Results in the figure show effect of the minor vs major allele; negative values indicate the minor allele has a lowering effect.

Figure 1, Panel A presents the association of genetically-predicted variation in apoB (based on SNPs in the ‘Lp(a)’ and ‘LDL’ clusters) with genetically-predicted variation in Lp(a) mass concentration. SNPs in the ‘Lp(a)’ cluster had a range of effect sizes (beta coefficients) for Lp(a) mass concentration and these were related in a clearly defined, proportionate gradient to genetically predicted effect sizes for apoB (as would be expected from the structure of the Lp(a) particle since each Lp(a) contains one apoB protein moiety). SNPs in the ‘LDL’ cluster gave a broad range of effect sizes for apoB but were not associated with variation in Lp(a) (beta coefficients for Lp(a) mass concentration were virtually zero). **Panel B** provides more detail of the relationship between genetically predicted Lp(a)-apoB and Lp(a) mass concentration for the SNPs in the Lp(a) cluster. There was excellent agreement in molar terms between the beta coefficients for these two independently derived exposures.

### Relationship of Lp(a) mass concentration to Lp(a)-apoB across ‘Lp(a)’ cluster polygenic score

Two polygenic scores (PGS) were calculated for each subject, the first based on a weighted sum of the number of apoB-raising alleles in the ‘Lp(a)’ cluster (i.e. Lp(a)-apoB), the second based on the number of apoB-raising alleles in the ‘LDL’ cluster (LDL-apoB). The whole cohort was then ranked both according to the ‘Lp(a)’ cluster PGS and according to the ‘LDL’ cluster PGS.

It was found first that mean measured Lp(a) mass concentrations (Randox assay) progressively increased across the ‘Lp(a)’ PGS ventiles, as expected (Online Figure 3A). Second, there was a linear, proportionate association between apoB concentration and Lp(a) mass concentration (both in nmol/L) across the PGS ventiles (Online Figure 3B). In theory, 1 mole of Lp(a)-apoB should be equivalent to 1 mole of Lp(a) mass concentration, and indeed the observed regression line was close to unity. This observation further supports the validity of Lp(a)-apoB as a genetic instrument to test association with CHD risk.

### Association of genetically predicted variation in apoB in the ‘Lp(a)’ and in the ‘LDL’ cluster with risk of a CHD event

**Figure 2:**
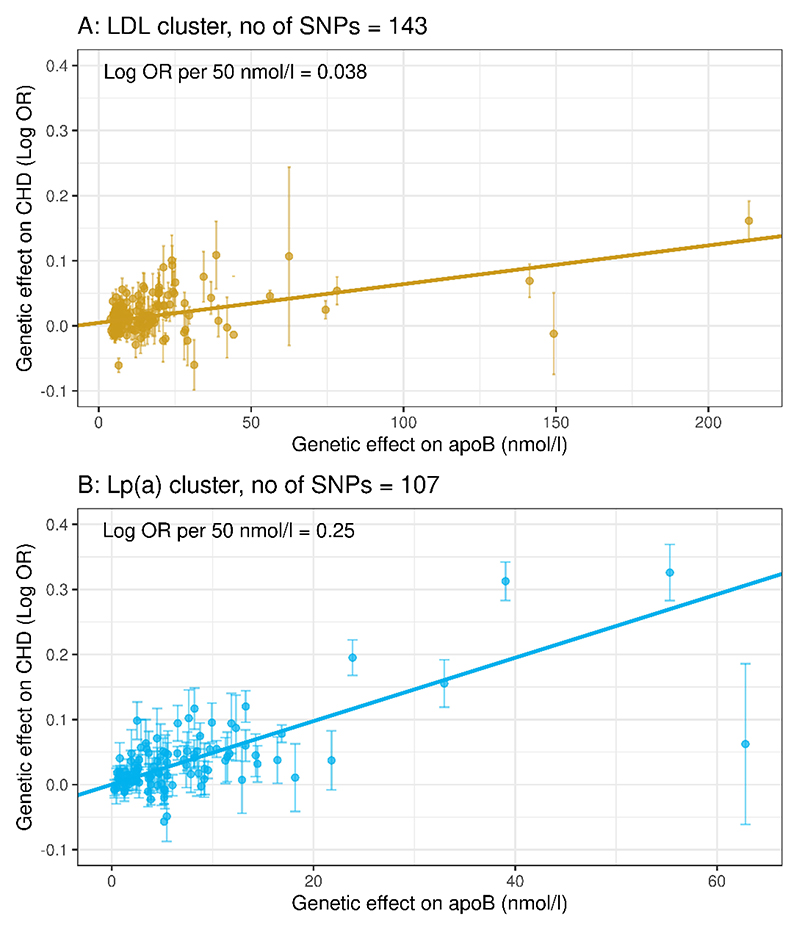
Relationship of CHD risk to LDL-apoB and Lp(a)-apoB. **Panel A:** Scatter plot of genetic effect sizes (beta-coefficients, with standard errors – see Figure legend 1 for further explanation) of LDL-apoB versus the genetic effect on CHD outcomes. **B:** Scatter plot of genetic effect sizes of Lp(a)-apoB against the genetic effect on CHD outcomes. The slope of the association in the ‘Lp(a)-cluster’ was greater than that in the ‘LDL’ cluster. The regression lines in A and B are calculated using inverse-variance weighting and thus the slope is interpreted as a log odds ratio (i.e. it is identical to the MR-model log odds ratio estimate).

Our aim was to compare directly the per-particle atherogenicity of Lp(a) versus LDL on the basis of a common constituent – apoB. It was observed that the gradient of association with CHD risk for genetically predicted variation apoB in Lp(a) (Figure 2 Panel B) was greater than for genetically predicted variation in apoB in LDL (**Panel A**). In Mendelian randomisation analyses, the respective odds ratios for CHD per 50 nmol/L higher apoB were 1.28 [95%CI: 1.24-1.33] for apoB in Lp(a) versus 1.04 [95%CI: 1.03-1.05] for apoB in LDL (Table 1). The CARDIoGRAMplusC4D case-control study was used in a replication analysis. In this data set, 98 of the Lp(a) cluster SNPs and 130 of the LDL cluster SNPs were present. Applying the Lp(a)-apoB and LDL-apoB beta coefficients derived using the UK Biobank to the CARDIoGRAMplusC4D data gave an CHD odds ratio (per 50 nmol/L higher apoB) for apoB in Lp(a) that was greater than that for apoB in LDL thereby confirming the findings from the UK Biobank (Table 1).

### Relative atherogenicity (CHD risk quotient) of Lp(a) versus LDL

A relative per-particle atherogenicity for Lp(a) compared to LDL was calculated for each SNP/data set (see Central Illustration). For the UK Biobank the relative atherogenicity (CHD risk quotient) per unit higher Lp(a)-apoB compared to LDL-apoB was 6.6 [95%CI: 5.1-8.8] (Table 2) whilst use of a previously published Lp(a) SNP set gave a risk quotient of 11.8 [95%CI:9.3-15.7] (see Online Figure 4 for a re-evaluation of the previously published SNPs set using the methodology described in the present paper). Likewise, applying the Lp(a)-apoB and LDL-apoB beta coefficients to the CARDIoGRAMplusC4D data gave a value of 3.8 [95%CI:2.7-5.4].

### Association of apoB with CHD risk using ‘Lp(a)’ and ‘LDL’ cluster polygenic scores

**Figure 3:**
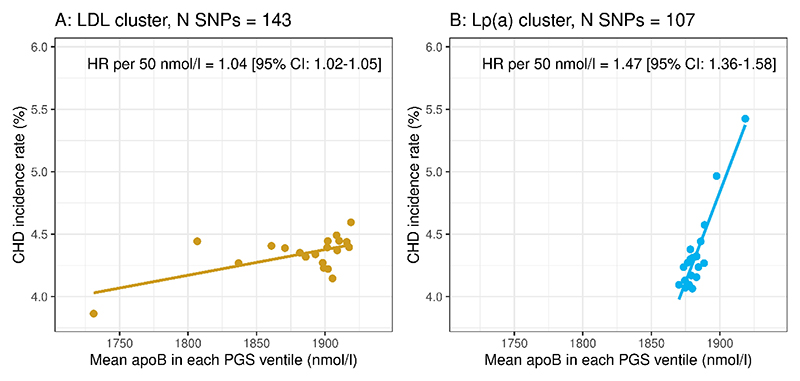
Relating apoB to CHD risk using ‘Lp(a)’ and ‘LDL’ PGSs. Two polygenic scores (PGS) based on the variants in each genetic cluster were constructed. Subjects were ranked by ‘LDL’ PGS and then divided into ventiles (**Panel A**). Separately, subjects were ranked into ventiles of ‘Lp(a)’ PGS (**Panel B**) (N≈24360 in each ventile). For each ventile in each of the PGS, the mean measured plasma apoB and observed CHD incidence rate were plotted against each other. For each PGS, the CHD hazard ratio per 50 nmol/l apoB was calculated by means of Cox proportional hazards modelling (adjusting for sex, BMI, age and systolic blood pressure).

In an alternative approach to determining the gradient of CHD risk per unit difference in genetically predicted Lp(a)-apoB and LDL-apoB, we examined the relationship of apoB to incident CHD risk by ventile of ‘Lp(a)’ PGS (Figure 3, Panel B), and by ventile of ‘LDL’ PGS (**Panel A**). Again, the gradient of association of apoB with CHD event rate was considerably steeper for cross-ventile differences in apoB associated with increasing ‘Lp(a)’ score than for differences in apoB associated with increasing ‘LDL’ score. Cox proportional hazards models gave hazard ratios per 50 nmol/L higher apoB of 1.47 [95%CI:1.36-1.58]) for the ‘Lp(a)’ cluster PGS vs 1.04 [95%CI:1.02-1.05]) for the ‘LDL’ cluster PGS.

### Evaluation of potential bias in Mendelian Randomisation and sensitivity analyses

Use of a polygenic approach to Mendelian Randomisation analysis can yield biased results if the assumption of the inverse-variance weighted approach is invalidated by SNPs having pleiotropic effects (that is, they impact CHD outcomes positively or negatively outside their effects on the exposure of interest – Lp(a) or LDL). Sensitivity analyses were conducted to determine the extent to which horizontal pleiotropy had biased the odds ratio estimates. As can be seen in Online Table 3, use of Mendelian Randomisation procedures that identify, and are tolerant of, potential SNP pleiotropic effects yielded odds ratios that were in close agreement with the data in Table 1.

Finally, we tested the possibility that choice of linkage disequilibrium r^2^ threshold during the pruning procedure may have influenced the results by repeating the GWAS SNP selection with r^2^<0.01. As seen in Online Figure 5 the gradient of apoB association with CHD risk in the ‘Lp(a)’ and ‘LDL’ clusters was close to that in Figure 2, and the calculated odds ratios were similar to those seen for the main analysis which used r^2^<0.1 (Table 1).

## Discussion

ApoB is present at one protein unit per particle in both Lp(a) and LDL, a key structural feature that allowed us to compare directly the relative atherogenicity (CHD risk quotient) of these lipoprotein species. Lp(a) particles, assessed on the basis of their component apoB, were found to have a several-fold (>6-fold in the UK Biobank) stronger association with CHD risk than LDL particles. This finding has important implications for understanding the CHD risk attributable to elevated Lp(a) levels, and for the design and interpretation of clinical outcome trials of Lp(a)-lowering.

There have been a number of previous attempts to quantify the atherogenicity of Lp(a) and to predict what change in its plasma concentration would be required to deliver a clinically useful reduction in CHD risk. Studies based on examining the association of variants in the *LPA* gene and in broader SNP sets associated with variation in plasma Lp(a) mass concentration have yielded a range of results. Burgess et al ^11^ calculated that, in a 5-year long clinical trial, a 101.5 mg/dL (about 220 nmol/L) lower Lp(a) would be required to reduce risk of cardiovascular events to the same degree as a 1.0 mmol/L lower LDL-C, i.e. by 22% (as estimated by meta-regression of mainly statin-based intervention studies ^23^). Others have reported that Lp(a) need only be lower by 55 mg/dL (about 120 nmol/L) to achieve this outcome ^12–14^. These results, which indicate that a substantial reduction of the absolute concentration of Lp(a) would be required to produce a clinically useful benefit may, as noted above, have been influenced among other issues by technical problems with the Lp(a) assays. In a previous analysis in the UK biobank, Welsh et al reported a significant independent association of measured Lp(a) particle concentrations with risk. They also explored the possibility that the association between Lp(a) and risk differed between subjects with high versus low LDL levels. They found that risk estimates differed but that the association with Lp(a) and risk was clinically significant regardless of the LDL levels ^24^.

By employing apoB as a common denominator in assessing the relationship of Lp(a) versus LDL to risk, we have potentially overcome several limitations. First, apoB assays are well standardised and less susceptible to bias than those for Lp(a), second apoB is relatively uniform in protein structure between individuals, and third since there is one mole of apoB per mole of Lp(a) or LDL, apoB-based odds ratios can be translated directly to per-particle risk. A further consideration in estimating the impact of a given degree of Lp(a) lowering on CHD risk is how to map a lifelong risk of higher Lp(a) (and conversely the lifelong benefit of a lower concentration) on to the timescale of a typical clinical trial. A 1 mmol/l higher life-time LDL-C is expected to result in a CHD odds ratio of 1.54; which translates into a 22 % risk reduction within the context of a 5-year clinical trial ^25^. Here we estimated that an 85 nmol/L (around 40 mg/dL) higher life-time Lp(a) results in a CHD odds ratio of 1.54. Thus, if drug-induced Lp(a)-lowering reduces risk in the same manner as LDL-C does, a lowering of 85 nmol/L Lp(a) is expected to decrease CHD risk by 22% within a 5-year clinical trial.

Our findings indicate that Lp(a) is indeed a highly atherogenic lipoprotein. The basis for this heightened atherogenic effect on CHD risk is not yet clear. It may be due, among other mechanisms, to the high content of oxidised phospholipids on Lp(a) that may stimulate inflammatory pathways involved in atherosclerosis, or to the structural similarity of apo(a) to plasminogen and hence its possible effects on clot stability ^26^. The quantitative estimates of the atherogenicity of Lp(a) generated in the present genetic study exceed those reported previously ^11–13^ and indicate that this lipoprotein is a viable target for intervention that is likely to yield a clinically useful risk reduction in a wider range of individuals than previously thought (relatively few individuals have a Lp(a) mass concentration in excess of 100 mg/dL (220nmol/l) as a starting level for Lp(a) lowering treatment) ^27^. The association of increased Lp(a) concentrations with risk of CHD is well established. Emerging results including recent meta-analysis studies indicate that Lp(a) is also an independent risk factor for stroke ^1, 27^, aortic valve stenosis ^1, 29–31^ and likely peripheral artery disease ^1, 31, 32^.

The findings of the present study have relevance for risk assessment in those with elevated Lp(a), setting thresholds for intervention to prevent disease outcomes, and for the design and interpretation of trials with newly developed Lp(a) lowering agents such as pelacarsen and olpasiran ^9, 10^. While we await the results of trials of Lp(a) as a target for intervention, it is useful to consider recent reports of outcome studies in which for the first time Lp(a) was reduced substantially by the drug under investigation. In FOURIER and Odyssey Outcomes, Lp(a) at baseline was a predictor of ongoing CHD risk in subjects with low LDL-C levels, and both evolocumab and alirocumab lowered Lp(a) by an average of 23% to 29% in addition to reducing LDL-C by 50-60% ^6, 7^. The higher atherogenicity of Lp(a) relative to LDL as reported in the present study helps explain why in both trials it was observed that the relative risk reduction on a PCSK9 inhibitor tended to be lower in subjects with the lowest baseline Lp(a) levels despite substantial LDL-C lowering, and conversely greater in subjects with the highest baseline Lp(a) who experienced the largest Lp(a) decrease. Further, in FOURIER the investigators estimated the relative risk reduction attributable to the lowering of Lp(a) as 15% per 25 nmol/L decrease ^6^. This value is of the same order as the predicted risk reduction from our analysis of the gradient between Lp(a)-apoB and CHD risk. It is noteworthy that the mean absolute decrease in Lp(a) mass concentration in ODYSSEY Outcomes was 5.0 mg/dl (approximately 12 nmol/L) while in FOURIER the median decrease was 11 nmol/L. Considering the earlier predicted responses to Lp(a) lowering ^1^, these small changes should have had little impact on relative risk reduction seen in the trials. However, the consistent observation in both outcome studies that a decrease in Lp(a) contributed significantly to the overall benefit suggests that the atherogenicity of the particle is greater, as indicated by our findings.

### Study limitations

Our experimental approach has both strengths and limitations. Since we focussed on the apoB component in Lp(a) in an analysis that did not select SNPs on the basis on their linkage to apo(a) size, we are, on the one hand, unable to distinguish differences in the association of Lp(a) particles carrying apo(a) isoforms of varying length (kringle 4 copy number) with CHD risk. On the other hand, our analysis should provide an aggregate result across Lp(a) particles which is relevant clinically since Lp(a) lowering agents are likely to be administered on the basis of total Lp(a) mass concentration rather than a specific apo(a) isoform abundance. Second, the SNPs used to determine genetically predicted variation in Lp(a) were all located in a relatively small region of chromosome 6, the *LPA* gene locus. While we attempted to exclude linked SNPs which might possibly inflate the risk estimate by adopting stringent thresholds for linkage disequilibrium SNP pruning, we cannot eliminate the possibility that a degree of linkage remained. Third, the study was conducted primarily in a Caucasian population and should be repeated in other ethnic groups. Fourth, the Lp(a) assay employed in the UK Biobank may have residual isoform dependency and underread at high Lp(a) levels. This assay feature may have influenced the measurement of high Lp(a) in this study. Further, the working range for the assay did not cover the full range of Lp(a) values seen in the population; about 10% of measurements were below the lower limit (<3.8nmol/l), and 6-7% were above the upper limit (>189nmol/l) of detection. This assay feature in theory may affect the detection of variants affecting Lp(a) levels, but it should be borne in mind that the main findings are based on genetically predicted variation in apoB. Finally, variation in Lp(a)-apoB and LDL-apoB were estimated from genetic analysis (the effect sizes of SNPs in the ‘Lp(a)’ and ‘LDL’ clusters on total apoB) and not measured specifically in biochemical assays. This apoB-based approach may be a particularly effective means of comparing Lp(a) association with risk across populations.

In our analysis we did not differentiate between subjects with or without CHD at baseline, and hence the risk estimates for Lp(a) versus LDL we generated incorporate both the primary and secondary prevention scenarios within the context of the UK Biobank population. However, current pharmaceutical interventions in both primary and secondary prevention settings may modify the association of Lp(a) with CHD risk. Only when the results of trials of Lp(a) lowering in individuals with and without CHD are reported will it become clear how these genetically determined risk estimates translate into clinical practice.

## Conclusions

This study provides evidence that apoB is a useful biomarker/exposure which allows direct comparison of the relative atherogenicity (CHD risk quotient) of Lp(a) versus LDL. Our results demonstrate that the strength of association of Lp(a) with CHD events was several-fold higher than that for LDL on a per-particle basis. In the ongoing Lp(a)-HORIZON study ^33^ which is designed to test the benefit of Lp(a) lowering with pelacarsen ^9^, a RNA-based (antisense oligonucleotide) agent which suppresses apo(a) synthesis, subjects were recruited on the basis of an elevated Lp(a) (outcome to be tested in subjects with levels ≥ 70 mg/dl (155 nmol/L) or ≥ 90 mg/dL (200 nmol/L)) and the expected decrease in Lp(a) is >80%. Thus, the anticipated decrease in Lp(a) of >150 nmol/L should, according to the findings of our study, generate a substantial and clinically meaningful decrease in risk over the planned length of the study, possibly of the order of 30-40%. On a population basis, it should be noted that Lp(a) levels are generally low and even with a higher per-particle atherogenicity, Lp(a) particles will make a much smaller contribution to overall risk compared to the more abundant LDL particles. However, in a sizeable minority of individuals with elevated Lp(a), the contribution of Lp(a) to CHD risk is considerably more significant.

## Supplementary Material

Supplementary Material

## Figures and Tables

**Central Illustration:** Relative atherogenicity of lipoprotein(a) and low-density lipoprotein particles. Causal estimates for the CHD risk per Lp(a) or LDL particle were compared by identifying genetic variants that specifically affected Lp(a) or LDL concentrations, and then quantifying their effect on apoB. The association of genetically predicted variation in apoB in Lp(a) with CHD events was then compared to that of genetically predicted variation in apoB in LDL using Mendelian Randomisation analysis. The atherogenicity (increase in CHD risk per unit change in particle concentration) for Lp(a)-apoB was about 6-fold greater than that for LDL-apoB.

**Table 1:** Genetically predicted variation in apoB and CHD risk in the ‘Lp(a)’ and ‘LDL’ clusters.

	No of SNPs	Odds Ratio per 50 nmol/l (95% CI)	P-value
**UK Biobank**			
LDL cluster	143	1.038 [1.029-1.048]	3.23×10^-17^
Lp(a) cluster	107	1.28 [1.24-1.33]	4.06×10^-47^
**CARDIoGRAMplusC4D**			
LDL cluster	130	1.041 [1.030-1.053]	3.72×10^-13^
Lp(a) cluster	98	1.17 [1.13-1.20]	4.53×10^-21^

For both analyses, effect sizes (beta coefficients) for the exposure (apoB) were calculated using the UK Biobank data set. Beta coefficients for the CHD outcome were estimated separately for the UK Biobank and for the CARDIoGRAMplusC4D cohorts. Odds ratios were determined by Mendelian randomisation analysis.

**Table 2:** Relative atherogenic potential of Lp(a)-apoB versus LDL-apoB as assessed by Mendelian randomisation modelling.

Cohort/ SNP set	LDL-apoB log OR per 50 nmol/L (SE)	LDL-apoB OR per 50 nmol/l	Lp(a)-apoB log OR per 50 nmol/L (SE)	Lp(a)-apoB OR per 50 nmol/l	Relative atherogenicity of Lp(a)-apoB compared to LDL-apoB(95% CI)
**UK Biobank present SNP set**	0.038 (0.0045)	1.038	0.25 (0.017)	1.28	6.6 (5.1-8.8)
**UK Biobank previously published Lp(a) SNP set** ^11^	0.038 (0.0045)	1.038	0.45 (0.028)	1.56	11.8 (9.3-15.7)
**CARDIoGRAMplusC4D**	0.040 (0.0056)	1.041	0.15 (0.016)	1.17	3.8 (2.7-5.4)

Point estimates of log odds ratios (log OR, with standard errors in parenthesis) are shown for (a) the UK Biobank using the GWAS derived SNP set in the present report, (b) the UK Biobank cohort using a previously published SNP set, and (c) and for the CARDIoGRAMplusC4D cohort using the SNP set from the present report. Relative atherogenic potential is calculated by dividing the Lp(a) log OR by the LDL-apoB log OR. Confidence intervals for this ratio were generated using a bootstrap procedure.

## Condensed Abstract

Lipoprotein(a) (Lp(a)) is recognised as a causal risk factor for atherosclerotic cardiovascular disease, but the strength of the association is not yet clear. This study, using a genetic approach, related the atherogenicity of Lp(a) to that of low-density lipoprotein (LDL) as a benchmark. Both lipoprotein particles contain a single apolipoprotein B (apoB) and we tested the association of genetically predicted variation in Lp(a)-apoB with risk of coronary heart disease compared to that of LDL-apoB. Lp(a) was about 6 times more atherogenic than LDL per unit change in apoB (that is per particle).

## Clinical Perspectives

### Competency in Medical Knowledge

Based on data in the U.K. Biobank, both LDL and lipoprotein (a) particles contain a single apolipoprotein B (apoB), yet on a per-apoB particle basis, Lp(a)-apoB is associated with about 6 times the risk of coronary atherosclerosis than LDL-apoB.

### Translational Outlook

Focussed biochemical, tissue/cell culture, and animal studies are needed to understand the mechanisms underlying the greater atherogenicity of Lp(a) compared to LDL particles.

## Abbreviations

apo(a): apolipoprotein (a)
apoB: apolipoprotein B
CVD: coronary heart disease
GWAS: genome-wide association studies
LDL-C: low density lipoprotein cholesterol
Lp(a): lipoprotein (a)
MR: Mendelian randomisation
PCSK9: proprotein convertase subtilisin/kexin type 9
PGS: polygenic scores
SNPs: single nucleotide polymorphisms
