# Supplementary material for: Lipoprotein(a) is Markedly More Atherogenic than LDL: An Apolipoprotein B-based Genetic Analysis
Source: J Am Coll Cardiol. Author manuscript; Available in PMC 2024 Oct 16. (PMC7616706; doi:10.1016/j.jacc.2023.10.039)
Supplement: Supplementary Material [file EMS193446-supplement-Supplementary_Material.pdf]

SUPPLEMENTAL ONLINE MATERIAL

Lipoprotein(a) is markedly more atherogenic than LDL - an apolipoprotein B-based genetic analysis in the UK Biobank

Content

Page	Element included
2-3	Online Expanded Rationale and Methodology.
4	Online Table 1. Definition of CHD outcomes
5-6	Online Table 2. SNPs with largest effect size in ‘LDL’ and ‘Lp(a) clusters
7	Online Table 3. Evaluation of potential bias in Mendelian randomisation (MR) analysis.
8	Online Figure 1. Flowchart of GWAS design and SNP selection
9	Online Figure 2. Association of measured Lp(a) mass concentration with apoB by ‘Lp(a)’ cluster polygenic score in ventiles based on subjects not on lipid lowering therapy.
10	Online Figure 3. Association of genetically-predicted apoB variation with CHD using Lp(a) SNPs from Burgess et al (LDL SNPs as in main analysis).
11	Online Figure 4. Selection of SNPs in ‘Lp(a)’ and ‘LDL’ clusters using a linkage disequilibrium threshold of r^2<0.01.
12	References

Online Expanded Rationale and Methodology.

The novel approach to determining the CHD risk associated with variation in Lp(a) adopted in this study warrants further explanation beyond that given in the main text. Our approach is summarised in the following schematic:

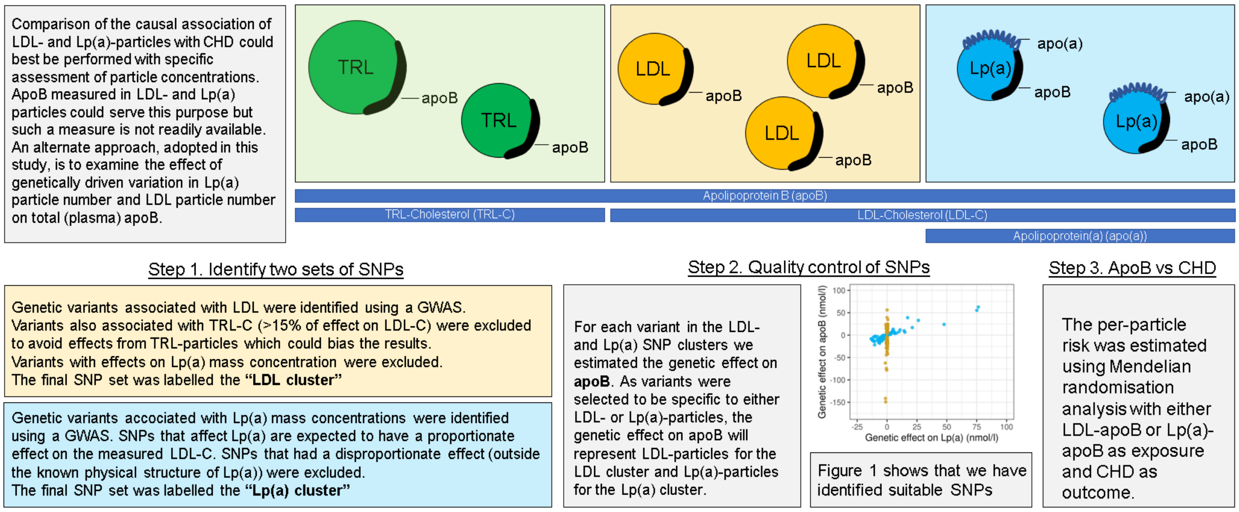

Our investigation depended on:

(i) the identification in a GWAS of SNPs associated specifically with Lp(a) as measured in the UK Biobank with the Randox assay (the ***'Lp(a)' SNP cluster***) (see **Online Table 2 and Figure 1**).

(ii) the identification of SNPs associated with LDL cholesterol (LDL-C) as measured in the UK Biobank cohort (the ***'LDL' SNP cluster***). Many SNPs linked to LDL-C are also associated with triglyceride rich lipoproteins (TRL) and their remnants[1] and SNPs which had a beta coefficient for TRL/remnant-cholesterol that was >15% of the beta coefficient for LDL-C (both in mmol/l) were excluded. Likewise, any SNPs associated with Lp(a) mass concentration were excluded from the 'LDL' cluster.

(iii) the quality of the apoB assay in the UK Biobank study.

Further:

(iv) it should be noted that our principal analyses were **not** based on relating the measurement of Lp(a) mass in the Randox assay to CHD risk and so any shortcomings in the assay of this lipoprotein were not a limitation in the interpretation of the results.

(v) The underlying rationale was that since both Lp(a) and LDL contain one apoB polypeptide (Lp(a) comprises a particle of LDL covalently bound to the apolipoprotein (a) protein), assessment of the genetically predicted variation in apoB using the 'Lp(a)' cluster SNPs or the 'LDL' cluster SNPs will be dependent on the accuracy and precision of the same biochemical assay for the determination of effect sizes, i.e. the assay of total plasma apoB.

Evaluation of genetically predicted variation in apoB in the 'Lp(a)' and 'LDL' SNP clusters.

For each of the 107 SNPs in the 'Lp(a)' cluster and the 143 SNPs in the 'LDL' cluster the beta coefficient (effect size) on apoB was estimated. In this way we could determine an effect size for ***Lp(a)-apoB*** and for ***LDL-apoB.***

By way of validation of the 'Lp(a)' and 'LDL' SNP clusters, we were able to demonstrate that:-

- SNP variants in the 'LDL' cluster had no effect on Lp(a) mass concentration (see main text **Figure 1**).
- SNPs in the 'Lp(a)' cluster had an effect size on apoB in Lp(a) that appeared equal numerically to the effect size on Lp(a) mass concentration when both were expressed in nmol/L (main text **Figure 1**). This is consistent with 1 mole of Lp(a) particles containing 1 mole of apoB.
- SNPs in the 'Lp(a)' cluster had effect sizes on LDL-C and apoB that were consistent with the known structure of Lp(a). That is, an LDL particle (with its contained cholesterol and apoB) is incorporated into Lp(a) (**Online Figure 2**).
- All the identified SNPs in the 'Lp(a)' cluster were located in the region of the *LPA* gene on chromosome 6.

Note this experimental design did not require the biochemical assay of apoB in Lp(a) particles. Rather, the SNP-based estimation of genetically predicted variation in Lp(a)-apoB and LDL-apoB permitted evaluation of the **causal** association of these variables with CHD outcomes. The relationship of genetically predicted variation in Lp(a)-apoB and LDL-apoB was assessed both for individual SNPs (main text **Figure 2**) and through use of a polygenic score for each cluster (main text **Figure 3**). Further, when considering the polygenic scores, the difference in mean measured apoB between ventiles using the 'LP(a)' cluster SNPs is attributable to the apoB component in Lp(a). Likewise, for the 'LDL' cluster SNPs, the difference between ventiles is due to the apoB in LDL.

Use of Lp(a) mass assay measurements

Apart from in the GWAS to identify SNPs associated with Lp(a) mass concentration, the other instances in which we employed the measured Lp(a) results were (i) as a sense check of the Lp(a)-apoB beta coefficients (main text **Figure 1B**) and (ii) the genetically predicted differences in Lp(a)-apoB relative to differences in Lp(a) mass concentration across ventiles of the polygenic score based on the 'Lp(a)' SNP cluster (**Online Figure 3**).

Online Table 1. Definition of CHD outcomes

**CHD outcome**		**Individuals, n = 487,202**
Non-fatal myocardial infarction (MI)	ICD 9 codes 410, 4110, 412, 42979<br>ICD 10 codes I21, I22, I23, I241, I252	Prevalent events n = 6,577<br>Incident events n = 17,356
Fatal MI	ICD 10 codes I21, I23, I241, I251, 1252, I253, I255-I259	Incident events n = 3,850
Coronary revascularisation	**Operational procedures**<br>Codes K501, K40-K44	Prevalent events n = 2,845<br>Incident events n = 3,571
Unique CHD outcomes	First event of above	Prevalent events n = 8,391<br>Incident events n = 20,792<br>Total events = 29,183

Incident events based on approximately 12 years of follow (as of January 2021).

Online Table 2. SNPs with largest effect size in 'LDL' and 'Lp(a)' clusters

SNP	Gene	Chr	Position	Beta apoB	Beta LDL-C	Beta Lp(a)
**LDL cluster**						
rs12721109	APOC2_APOC4	19	45447221	-0.1173	-0.3467	-0.96
rs11083761	NKPD1_MARK4	19	45655636	-0.08209	-0.2383	-1.169
rs76560105	CBLC	19	45299199	-0.07773	-0.2247	-1.34
rs139659653	NPC1L1	7	44578747	-0.03434	-0.1509	-1.44
rs62119261	IGSF23_CEACAM22P	19	45122043	-0.04299	-0.1263	-0.3006
rs11881756	CEACAM16_BCL3	19	45220896	-0.04093	-0.1207	-0.5626
rs148790687	DNM2	19	10883157	-0.02434	-0.103	-0.5396
rs56315738	SMARCA4_LDLR	19	11175823	-0.02315	-0.08902	0.4909
rs405509	APOE_TOMM40	19	45408836	0.03089	0.08451	0.03189
rs505151	PCSK9	1	55529187	0.02159	0.08037	-0.02321
rs74257940	LDLR	19	11214475	0.01892	0.07496	0.6599
rs62116988	KCNN4_LYPD5	19	44296689	-0.02026	-0.06204	-0.05688
rs41302083	PSRC1	1	109824680	-0.02119	-0.05995	0.03732
rs1800961	HNF4A	20	43042364	-0.01017	-0.05591	-0.5274
rs115357389	GDF7_HS1BP3	2	20863092	-0.01542	-0.05489	-0.2616
rs1229984	ADH1B	4	100239319	-0.01091	-0.05444	0.4704
rs78755596	ABO_OBP2B	9	136124590	0.01085	0.05316	-0.7721
rs111282584	SPC24	19	11262229	0.01319	0.05283	-1.035
rs77231091	MAP2K6_KCNJ16	17	67547201	0.01282	0.05191	0.2098
rs117490455	SMARCA4	19	11170677	0.01325	0.0508	0.2682
rs11244084	SURF6_ABO	9	136191010	0.01077	0.0495	0.1267
rs62116303	ZNF229_ZNF180	19	44957310	-0.01632	-0.04908	-0.2577
rs76797241	ZNF223_ZNF222	19	44554289	-0.01721	-0.04739	0.3756
rs12609269	CEACAM20	19	45033285	-0.01549	-0.04652	-0.5303
rs10205003	APOB_LOC645949	2	21482439	-0.01601	-0.04626	-0.4984
**Lp(a) cluster**						
rs8177505	SLC22A2	6	160258624	0.03455	0.1257	76.35
rs3918291	SLC22A3	6	160828142	0.03043	0.1197	75.09
rs117446263	SLC22A3	6	160847571	0.01313	0.04454	47.74
rs146534110	SLC22A1	6	160578069	0.01813	0.07418	26.2
rs10945656	SLC22A2_SLC22A1	6	160635886	0.00927	0.03313	21.11
rs2282143	SLC22A1	6	160557643	0.02147	0.0813	17.21
rs73020718	FLJ27255_SOD2	6	160032984	0.003599	0.01211	14.9
rs6415084	LPA	6	160980330	0.005354	0.01945	14.26
rs41267809	LPA	6	160953642	-0.004511	-0.01597	-13.3
rs143431368	LPA	6	160969693	-0.004652	-0.02006	-13.08
rs555754	SLC22A3	6	160769423	-0.006421	-0.02238	-12.31
rs35509017	PLG_MAP3K4	6	161217544	-0.006298	-0.02219	-11.54
rs41259144	LPA	6	161022107	-0.00247	-0.01394	-11.43
rs41272114	LPA	6	161006077	-0.007306	-0.02414	-11.41
rs75975688	AGPAT4_PARK2	6	161701726	0.006523	0.02313	10.89
rs118001500	SLC22A2_SLC22A3	6	160683206	-0.007847	-0.0249	-10.43
rs74907759	SLC22A3	6	160857193	-0.01198	-0.04092	-10.42

rs58364041	SLC22A3_LPAL2	6	160874677	-0.002012	-0.009743	-10.38
rs117332585	SOD2_FNDC1	6	159857839	0.004119	0.01268	9.99
rs4252129	PLG	6	161152905	-0.001074	-0.008043	-9.784
rs3798158	SLC22A2	6	160646222	-0.004815	-0.02033	-8.178
rs35589108	TCP1	6	160207845	-0.006199	-0.01968	-8.148
rs1937475	PLG_MAP3K4	6	161315724	0.002591	0.007853	8.017
rs783147	PLG	6	161137990	-0.001907	-0.007753	-7.713
rs74334585	LPA	6	161011907	-0.009035	-0.03293	-7.12

Abbreviations: Chr, chromosome; beta, beta coefficient.

The beta-coefficients can be interpreted as the expected change in the exposure (apoB, LDL-C or Lp(a)) by one extra effect-allele. Negative beta-coefficients indicate a lipoprotein-lowering effect of the SNP (minor/less frequent allele vs major allele) and positive values indicate a lipoprotein-raising effect.

Online Table 3. Evaluation of potential bias in Mendelian randomisation (MR) analysis.

Method	Cluster	OR per 50 nmol/l	CI low	CI high	P-value
Simple median	LDL	1,051	1,035	1,066	9,6E-11
Weighted median	LDL	1,038	1,028	1,049	2,9E-13
Penalized weighted median	LDL	1,039	1,028	1,049	2,8E-13
IVW	LDL	1,038	1,029	1,048	3,2E-17
Penalized IVW	LDL	1,044	1,035	1,052	4,1E-25
Robust IVW	LDL	1,040	1,032	1,048	3,3E-22
Penalized robust IVW	LDL	1,043	1,037	1,050	9,2E-38
MR-Egger	LDL	1,030	1,019	1,042	2,5E-07
(intercept)	LDL	1,00013	1,00002	1,00024	0,026
Simple median	Lp(a)	1,27	1,19	1,35	1,5E-13
Weighted median	Lp(a)	1,28	1,22	1,34	8,9E-26
Penalized weighted median	Lp(a)	1,26	1,21	1,33	5,9E-23
IVW	Lp(a)	1,28	1,24	1,33	4,1E-47
Penalized IVW	Lp(a)	1,27	1,24	1,31	1,2E-64
Robust IVW	Lp(a)	1,28	1,22	1,34	1,8E-26
Penalized robust IVW	Lp(a)	1,27	1,23	1,31	6,5E-51
MR-Egger	Lp(a)	1,29	1,23	1,34	<1E-100
(intercept)	Lp(a)	1,00	1,00	1,00	0,89

The close agreement of causal estimates across the statistical approaches provides a high degree of confidence in the application of the inverse-variance weighted (IVW) method in Mendelian randomisation models used in the present analysis. Briefly, in addition to the IVW method there are several methods or weighting-schemes that are designed to offer robustness against outlying genetic instruments. The median methods (simple, weighted and penalized weighted) all are robust methods when a minority of genetic instruments are outliers or invalid instrumental variables. The robust IVW implements a robust regression method instead of the standard linear regression method. Penalized robust IVW applies a penalty to down-weight outliers. Lastly, the MR-Egger tests for pleiotropic effects by introducing an intercept term. For further information, see the documentation to the MendelianRandomization R package (https://cran.r-project.org/web/packages/MendelianRandomization/MendelianRandomization.pdf). For each analysis, results are expressed as an odds ratio (OR) and 95% confidence interval (CI).

Online Figure 1. Flowchart of GWAS design and SNP selection

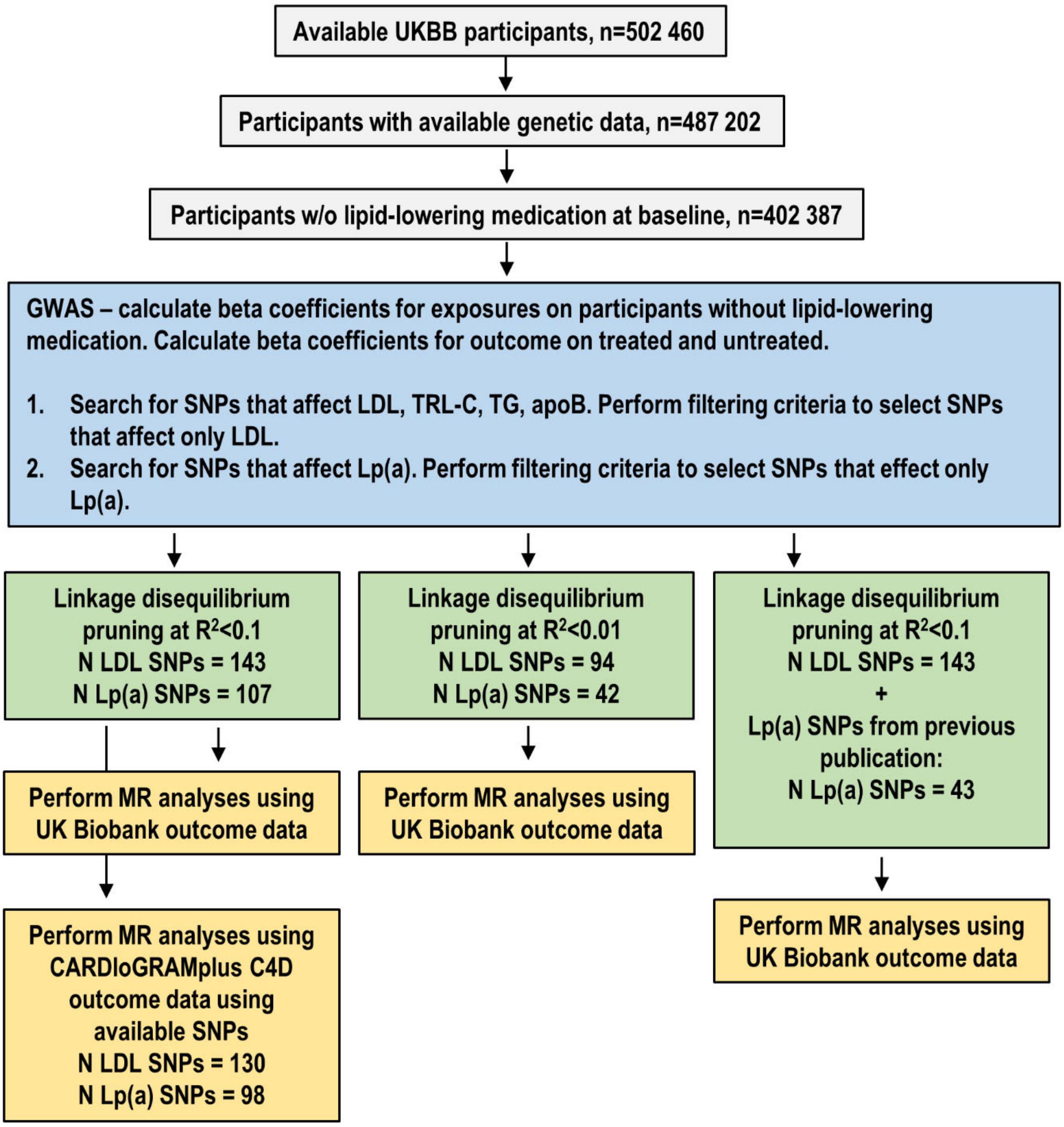

Online Figure 2. Relationship of genetically predicted variation in apoB with genetically predicted variation in LDL cholesterol (LDL-C) for SNPs in the 'Lp(a)' and 'LDL' clusters.

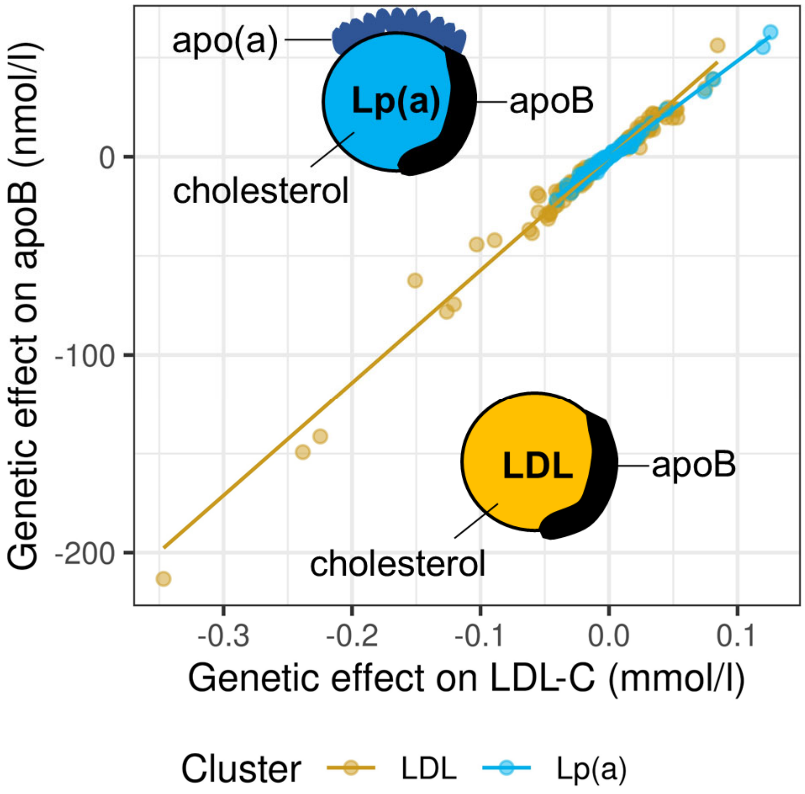

Cholesterol in Lp(a) particles is included in the measurement of LDL-C, and apoB in Lp(a) particles is included in the measurement of total plasma apoB (however, in most subjects the contribution of Lp(a) to total LDL-C and apoB is small due to the low Lp(a) mass concentration). Since an Lp(a) particle incorporates an LDL particle, then in theory the ratio of cholesterol to apoB in Lp(a) should equal the ratio of cholesterol to apoB in LDL particles.

This is what we observed. The figure above shows that the relationship of effect sizes for LDL-C and apoB for the 107 SNPs in the 'Lp(a)' cluster (due to variation in Lp(a) particle number) was the same as the relationship of effect sizes for LDL-C and apoB for the 143 SNPs in the 'LDL' cluster (due to variation in LDL particle number).

This finding indicates that the SNPs in the 'Lp(a)' cluster were providing a genetically driven variation in Lp(a) particle number that was in line with the known structural properties of Lp(a). Further, on the basis of this data it was considered unlikely that the SNPs in the 'Lp(a)' cluster were influencing any other lipoprotein species.

Online Figure 3. Association of measured Lp(a) mass concentration with apoB by 'Lp(a)' cluster polygenic score in ventiles based on subjects not on lipid lowering therapy.

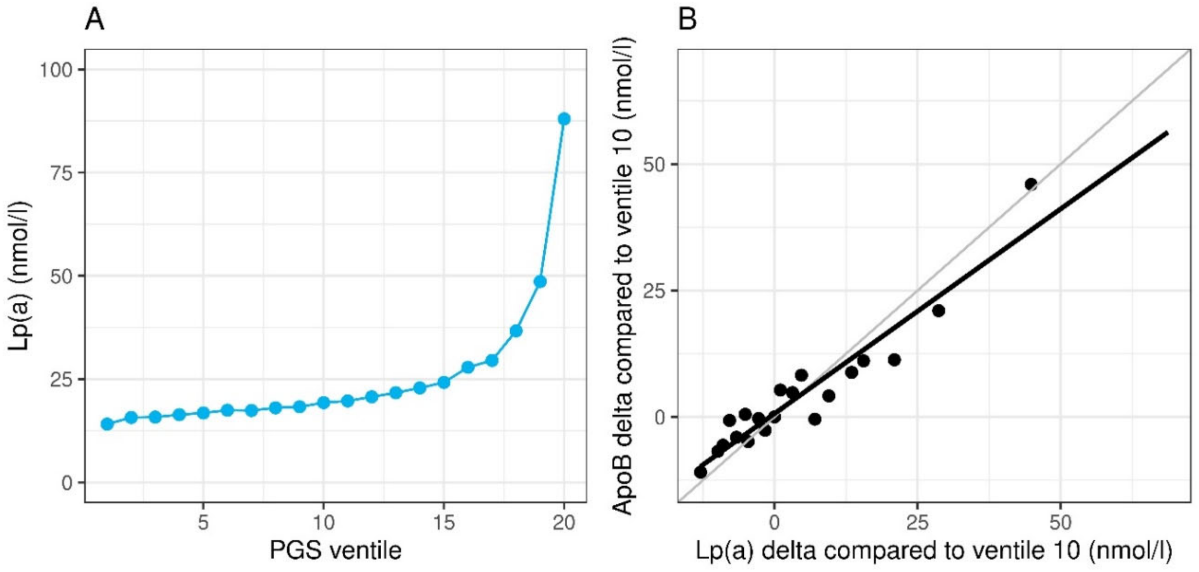

Untreated subjects in the UK Biobank cohort (n= 415 535) were ranked by 'Lp(a)'-PGS and then divided into ventiles (20[ths,] i.e. with 20776 or 20777 subjects in each ventile). **Panel A** presents the mean measured Lp(a) mass concentration in nmol/L (Randox assay) for each ventile. In **Panel B**, the mean plasma Lp(a) and mean plasma apoB and the subsequent difference (delta) from ventile 10 (used as reference) are plotted against each other. The differences in apoB between ventiles when subjects are ranked in this way is due to the higher level of Lp(a) as shown in **Figure 1** in the main text. In theory, since one apoB is present per particle, then the two measures should show agreement, that is 1 nmol/L of Lp(a) should equal 1 nmol/L of apoB. The data presented in **Panel B** supports this contention.

Online Figure 4. Association of genetically-predicted apoB variation with CHD using Lp(a) SNPs from Burgess et al[2] ('LDL' cluster SNPs as in main analysis).

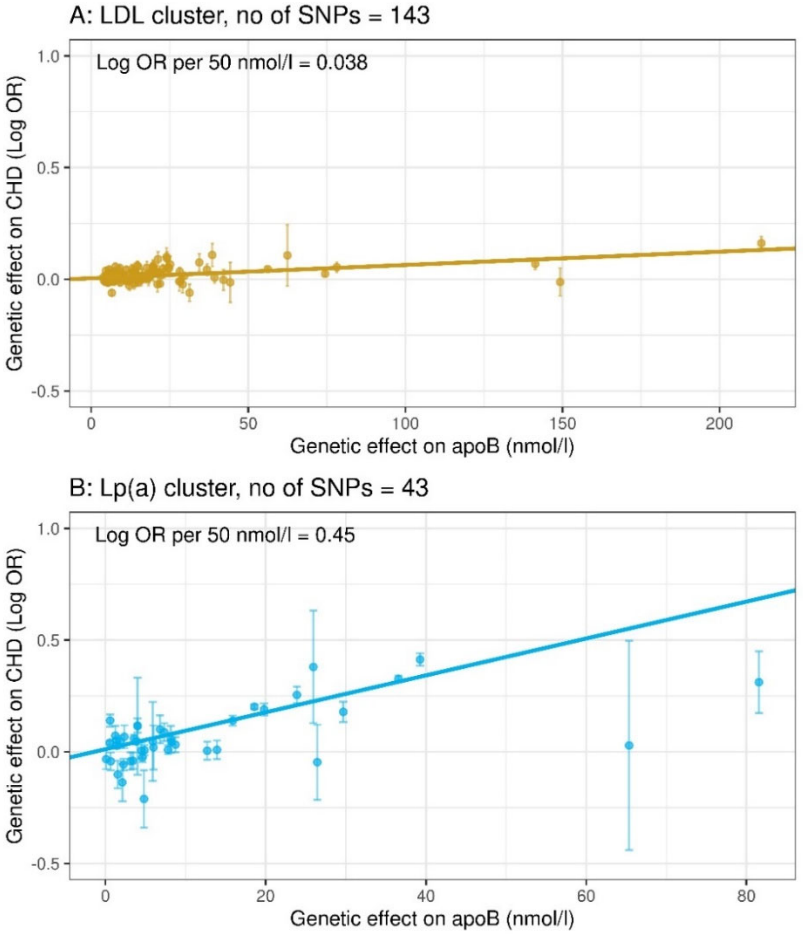

	N SNPs	LOG OR per 50 nmol/l	LOG OR CI low	LOG OR CI high	OR per 50 nmol/l	OR CI low	OR CI high	P-value
LDL cluster	143	0.0378	0.029	0.0465	1.04	1.03	1.05	3.23e-17
Lp(a) SNPs (Burgess et al[2])	43	0.447	0.393	0.502	1.56	1.48	1.65	2.81e-58

A: Scatter plot of effect sizes (beta-coefficients) of LDL-apoB against the genetic effects on CHD outcomes. B: Scatter plot of effect sizes of Lp(a)-apoB against the genetic effects on CHD outcomes. The slope for the 'Lp(a)' cluster was found to be greater than for the slope for the 'LDL' cluster. Table shows MR-model results produced by modelling the genetic instruments plotted in A (the 'LDL' cluster) and in B (the 'Lp(a)' cluster). MR model results for the Lp(a) SNP set was further adjusted for internal correlation, which only marginally affected the estimate (data not shown). Note that in the present study SNPs in the 'Lp(a)' cluster were identified in an 'agnostic' procedure in which SNP selection was not based on the strength of the association, only on its presence. In the previous study, SNPs for Lp(a) were 'selected' based on an algorithm that added variants only if they improved prediction of Lp(a) levels[2]. The main difference between the two SNP sets lies in the inclusion in the main analysis of SNP with weaker effect sizes in the 10-40 nmol/L range of genetically-predicted apoB (compare **Panel B** above with main text **Figure 2B**).

Online Figure 5. Selection of SNPs in 'Lp(a)' and 'LDL' clusters using a linkage disequilibrium threshold of r^2<0.01.

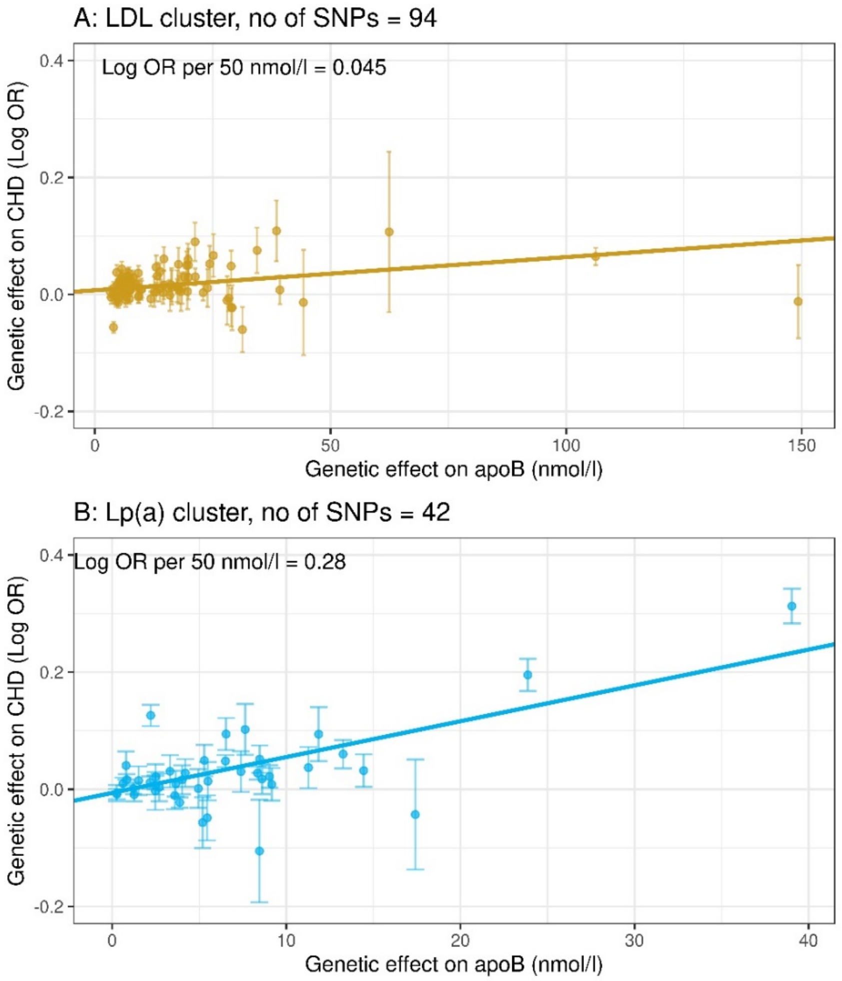

	N SNPs	LOG OR per 50 nmol/l	LOG OR CI low	LOG OR CI high	OR per 50 nmol/l	OR CI low	OR CI high	P-value
LDL cluster	94	0.0447	0.031	0.0583	1.05	1.03	1.06	1.43e-10
Lp(a) cluster	42	0.285	0.212	0.358	1.33	1.24	1.43	2.11e-14

A: Scatter plot of SNP effect sizes (beta-coefficients) for LDL-apoB against the genetic effects on CHD outcomes. **B:** Scatter plot of SNP effect sizes for Lp(a)-apoB against the genetic effects on CHD outcomes. The slope of the 'Lp(a)' cluster was found to be greater than for the slope of the 'LDL' cluster. Table shows the findings for the MR-model produced by modelling the genetic instruments plotted in **A** (the 'LDL' cluster) and in **B** (the 'Lp(a)' cluster).

REFERENCES

1. Björnson E, Adiels M, Taskinen MR, Burgess S, Rawshani A, Borén J and Packard CJ. Triglyceride-rich lipoprotein remnants, low-density lipoproteins, and risk of coronary heart disease: a UK Biobank study, *Eur Heart J*. 2023. In Press.
2. Burgess S, Ference BA, Staley JR, et al. Association of LPA Variants With Risk of Coronary Disease and the Implications for Lipoprotein(a)-Lowering Therapies: A Mendelian Randomization Analysis. *JAMA Cardiol*. 2018;3:619-627.
